# Fragmented QRS Is Independently Predictive of Long-Term Adverse Clinical Outcomes in Asian Patients Hospitalized for Heart Failure: A Retrospective Cohort Study

**DOI:** 10.3389/fcvm.2021.738417

**Published:** 2021-11-11

**Authors:** Jeffrey Shi Kai Chan, Jiandong Zhou, Sharen Lee, Andrew Li, Martin Tan, Keith Sai Kit Leung, Kamalan Jeevaratnam, Tong Liu, Leonardo Roever, Ying Liu, Gary Tse, Qingpeng Zhang

**Affiliations:** ^1^Cardiovascular Analytics Group, Laboratory of Cardiovascular Physiology, Hong Kong, Hong Kong SAR, China; ^2^School of Data Science, City University of Hong Kong, Hong Kong, Hong Kong SAR, China; ^3^Faculty of Science, University of Calgary, Calgary, AB, Canada; ^4^Department of Immunology, University of Toronto, Toronto, ON, Canada; ^5^Emergency Medicine Unit, Li Ka Shing Faculty of Medicine, The University of Hong Kong, Hong Kong, Hong Kong SAR, China; ^6^Faculty of Health and Medical Sciences, University of Surrey, Guildford, United Kingdom; ^7^Tianjin Key Laboratory of Ionic-Molecular Function of Cardiovascular Disease, Department of Cardiology, Tianjin Institute of Cardiology, Second Hospital of Tianjin Medical University, Tianjin, China; ^8^Departamento de Pesquisa Clinica, Universidade Federal de Uberlandia, Uberlandia, Brazil; ^9^Heart Failure and Structural Cardiology Division, The First Affiliated Hospital of Dalian Medical University, Dalian, China; ^10^Kent and Medway Medical School, Canterbury, United Kingdom

**Keywords:** fragmented QRS, heart failure, Asian, ventricular arrhythmia, sudden cardiac death, myocardial fibrosis

## Abstract

**Background:** Fragmented QRS (fQRS) results from myocardial scarring and predicts cardiovascular mortality and ventricular arrhythmia (VA). We evaluated the prevalence and prognostic value of fQRS in Asian patients hospitalized for heart failure.

**Methods and Results:** This was a retrospective cohort study of adult patients hospitalized for heart failure between 1st January 2010 and 31st December 2016 at a tertiary center in Hong Kong. The baseline ECG was analyzed. QRS complexes (<120 ms) with fragmented morphology in ≥2 contiguous leads were defined as fQRS. The primary outcome was a composite of cardiovascular mortality, VA, and sudden cardiac death (SCD). The secondary outcomes were the components of the primary outcome, myocardial infarction, and new-onset atrial fibrillation. In total, 2,182 patients were included, of whom 179 (8.20%) had fQRS. The follow-up duration was 5.63 ± 4.09 years. fQRS in any leads was associated with a higher risk of the primary outcome (adjusted hazard ratio (HR) 1.428 [1.097, 1.859], *p* = 0.001), but not myocardial infarction or new-onset atrial fibrillation. fQRS in >2 contiguous leads was an independent predictor of SCD (HR 2.679 [1.252, 5.729], *p* = 0.011). In patients without ischaemic heart disease (*N* = 1,396), fQRS in any leads remained predictive of VA and SCD (adjusted HR 3.526 [1.399, 8.887], *p* = 0.008, and 1.873 [1.103, 3.181], *p* = 0.020, respectively), but not cardiovascular mortality (adjusted HR 1.064 [0.671, 1.686], *p* = 0.792).

**Conclusion:** fQRS is an independent predictor of cardiovascular mortality, VA, and SCD. Higher fQRS burden increased SCD risk. The implications of fQRS in heart failure patients without ischaemic heart disease require further studies.

## Introduction

First described by Boineau and Cox in 1973, fragmented QRS (fQRS) is the manifestation of myocardial scarring on 12-lead surface electrocardiogram (ECG) (1, 2). Though initially described in the context of ischaemic heart disease (IHD*)*, fQRS has been observed in other conditions where myocardial scarring or fibrosis is present, such as hypertrophic cardiomyopathy and cardiac sarcoidosis (2, 3). The presence of fQRS has been shown to be predictive of all-cause mortality and ventricular arrhythmia (VA) (4), and several morphological criteria and classification systems have been set forth by a number of research groups, including those by Das et al. and Haukilahti et al. (2, 5). While fQRS has been shown to be predictive of adverse cardiovascular outcomes in patients with heart failure (4), most studies have focused on either acute outcomes of hospitalized patients, or long-term outcomes of ambulatory patients (6, 7). With the rising prevalence of heart failure in Asia (8, 9), there is an ever greater need for good prognostic markers in Asian patients with heart failure. As data on the prevalence and long-term prognostic power of fQRS in Asian patients hospitalized with heart failure are lacking, we aimed to bridge this gap in evidence with the current study.

## Materials and Methods

This was a retrospective cohort study approved by The Joint Chinese University of Hong Kong—New Territories East Cluster Clinical Research Ethics Committee. All patients aged ≥18 years old who were hospitalized for heart failure, as identified using relevant ICD-9 codes, between 1st January 2010 and 31st December 2016 at a single tertiary center in Hong Kong were included. Patients who had wide QRS (≥120 ms) on the first ECG recorded during index hospitalization, missing primary outcome data, and those who did not have any ECG done during the index hospitalization were excluded. The patients were identified using the Clinical Data Analysis and Reporting System (CDARS), a territory-wide database that centralizes patient information from local hospitals and ambulatory and outpatient facilities. Mortality data were obtained from the Hong Kong Death Registry, a government registry with the registered death records of all Hong Kong citizens linked to CDARS.

Patient demographics, prior comorbidities (as identified by ICD-9 codes [Supplementary Table 1; anemia was additionally identified by a baseline hemoglobin level <13 g/dL (males) or <12 g/dL (females)], and baseline medication usage were extracted. The baseline Charlson comorbidity index was calculated to reflect comorbid statuses. The baseline ECG obtained on the first heart failure admission was selected for automated analysis. The paper speed of all ECG performed in public institutions in Hong Kong has been standardized to 25 mm/s, which therefore applies to all ECG analyzed in this study. Automated measurements were performed on digital ECG tracings using the Philips ECGVue program (Standard Edition), with the ECG waveform data captured at a sample rate of 4 MHz and reduced to 500 samples per second with 5 μV resolution. We defined fQRS using the criteria set forth by Das et al., where fQRS was defined as the presence of an additional R wave (R'), notching in the nadir of the S wave, or presence of more than two R' waves in at least two contiguous leads within any myocardial territory (anterior, inferior, or lateral) with QRS duration of <120 ms (5). QRS complexes with fragmented morphology in a single lead were not classified as fQRS.

The primary outcome was a composite of cardiovascular mortality, VA, and SCD. The secondary outcomes were the individual components of the primary composite outcome, myocardial infarction (MI), and new-onset atrial fibrillation (AF). All patients were followed up till 31st December 2019. All event occurrences were identified using ICD-9 codes.

All continuous variables were expressed as mean ± standard deviation and compared using Student's *t*-test. Dichotomous variables were compared using Fisher's exact test. All outcomes were analyzed by univariate and multivariate Cox regression adjusting for baseline comorbidities that significantly predicted the outcomes. Hazard ratios (HR) were used as the summary statistic. Subgroup analyses were done to assess the prognostic power of fQRS in patients with and without ischaemic heart disease, respectively. The impact of fQRS burden in patients with fQRS was also explored by comparing those who only have two leads with fQRS morphology against those who have more than two such leads. All *p*-values were two-sided, and *p* < 0.05 was considered significant. All statistical analyses were performed using SPSS software version 25.0 (IBM Corp, New York, USA). The raw data supporting the conclusions of this article will be made available on reasonable request to any of the corresponding authors, without undue reservation.

## Results

**Figure 1:**
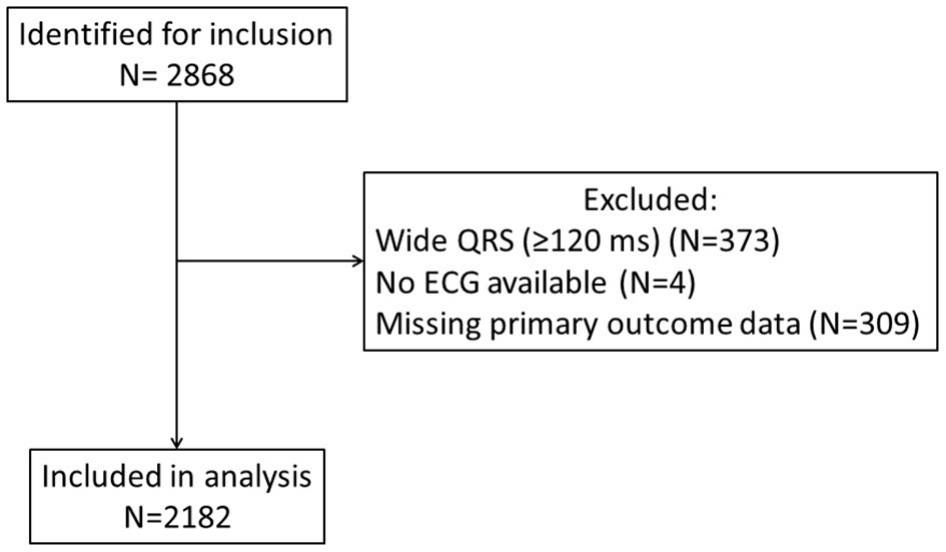
Flow chart summarizing the inclusion and exclusion of patients.

In total, 2,868 patients fulfilled the inclusion criteria. After excluding patients with missing ECG (*N* = 4), missing primary outcome data (*N* = 309), and wide QRS complex (*N* = 373), 2,182 patients were included in the analysis (Figure 1). We identified fQRS in 179 patients (8.20%), more of whom were males than those without fQRS [98 of 179 (54.7%) vs. 934 of 2003 (46.6%), *p* = 0.042], but were not significantly different in age (75.4 ± 14.8 vs. 74.4±13.5 years, *p* = 0.403) or Charlson comorbidity index (4.91 ± 2.29 vs. 5.14 ± 2.39, *p* = 0.211); 1,588 (72.8%) had known heart failure prior to the index hospitalization. Patients with fQRS had significantly higher rates of chronic renal diseases (*p* = 0.011) but lower rates of anemia (*p* = 0.020). Other baseline characteristics were not significantly different between the two cohorts (Table 1). Follow-up durations were similar between cohorts (5.53 ± 4.21 vs. 5.64 ± 4.08 years, *p* = 0.733), without any loss to follow-up. The primary composite outcome was met in 566 (25.9%) patients, while the secondary outcome of cardiovascular mortality was met in 419 (19.2%) patients, VA in 56 (2.6%) patients, SCD in 212 (9.7%) patients, MI in 353 (16.2%) patients, and new-onset AF in 829 (38.0%) patients.

**Table 1:** Baseline characteristics and follow-up durations of included patients.

	**No fQRS (*N* = 2,003)**	**fQRS present (*N* = 179)**	***P*-value**
Follow-up duration, years	5.64 ± 4.08	5.53 ± 4.21	0.733
**Demographics**			
Male. *N* (%)	934 (46.6)	98 (54.7)	0.042
Age, years	74.4 ± 13.5	75.4 ± 14.8	0.403
**Comorbidities**			
Charlson comorbidity index	5.14 ± 2.39	4.91 ± 2.29	0.211
Anemia, *N* (%)	785 (39.2)	54 (30.2)	**0.020**
Known heart failure prior to index admission, *N* (%)	1,469 (73.3)	119 (66.5)	0.054
Diabetes mellitus, *N* (%)	616 (30.8)	50 (27.9)	0.447
Chronic renal diseases, *N* (%)	277 (13.8)	13 (7.26)	**0.011**
Hypertension, *N* (%)	918 (45.8)	69 (38.5)	0.071
Ischaemic heart disease, *N* (%)	711 (35.5)	75 (41.9)	0.089
Myocardial infarction, *N* (%)	185 (9.2)	19 (10.6)	0.505
Prior ventricular arrhythmia, *N* (%)	21 (1.0)	5 (2.8)	0.056
Prior sudden cardiac death, *N* (%)	22 (1.1)	4 (2.2)	0.159
Atrial fibrillation, *N* (%)	370 (18.5)	41 (22.9)	0.162
Stroke/TIA, *N* (%)	264 (13.2)	18 (10.1)	0.294
**Medications**			
ACEI/ARB, *N* (%)	1,016 (50.7)	95 (53.1)	0.585
Beta-blockers, *N* (%)	964 (48.1)	92 (51.4)	0.435
Diuretics, *N* (%)	755 (37.7)	69 (38.5)	0.810
Non-dihydropyridine calcium channel blockers, *N* (%)	749 (37.4)	55 (30.7)	0.089
Nitrates, *N* (%)	518 (25.8)	57 (31.8)	0.092
Statins and fibrates, *N* (%)	621 (31.0)	51 (28.5)	0.554
Anticoagulants, *N* (%)	320 (16.0)	33 (18.4)	0.397
Antiplatelets, *N* (%)	798 (39.8)	74 (41.3)	0.691
Sodium channel-blocking antiarrhythmics, *N* (%)	6 (0.300)	1 (0.562)	0.451
Potassium channel-blocking antiarrhythmics, *N* (%)	56 (2.80)	3 (1.68)	0.478
Dihydropyridine calcium channel blockers, *N* (%)	174 (8.69)	16 (8.94)	0.890
Digoxin, *N* (%)	174 (8.69)	21 (11.7)	0.172

*ACEI, angiotensin-converting-enzyme inhibitors; ARB, angiotensin II receptor blockers; fQRS, fragmented QRS*.

*Statistically significant results were highlighted in bold*.

### Prognostic Value of the Presence of fQRS

**Figure 2:**
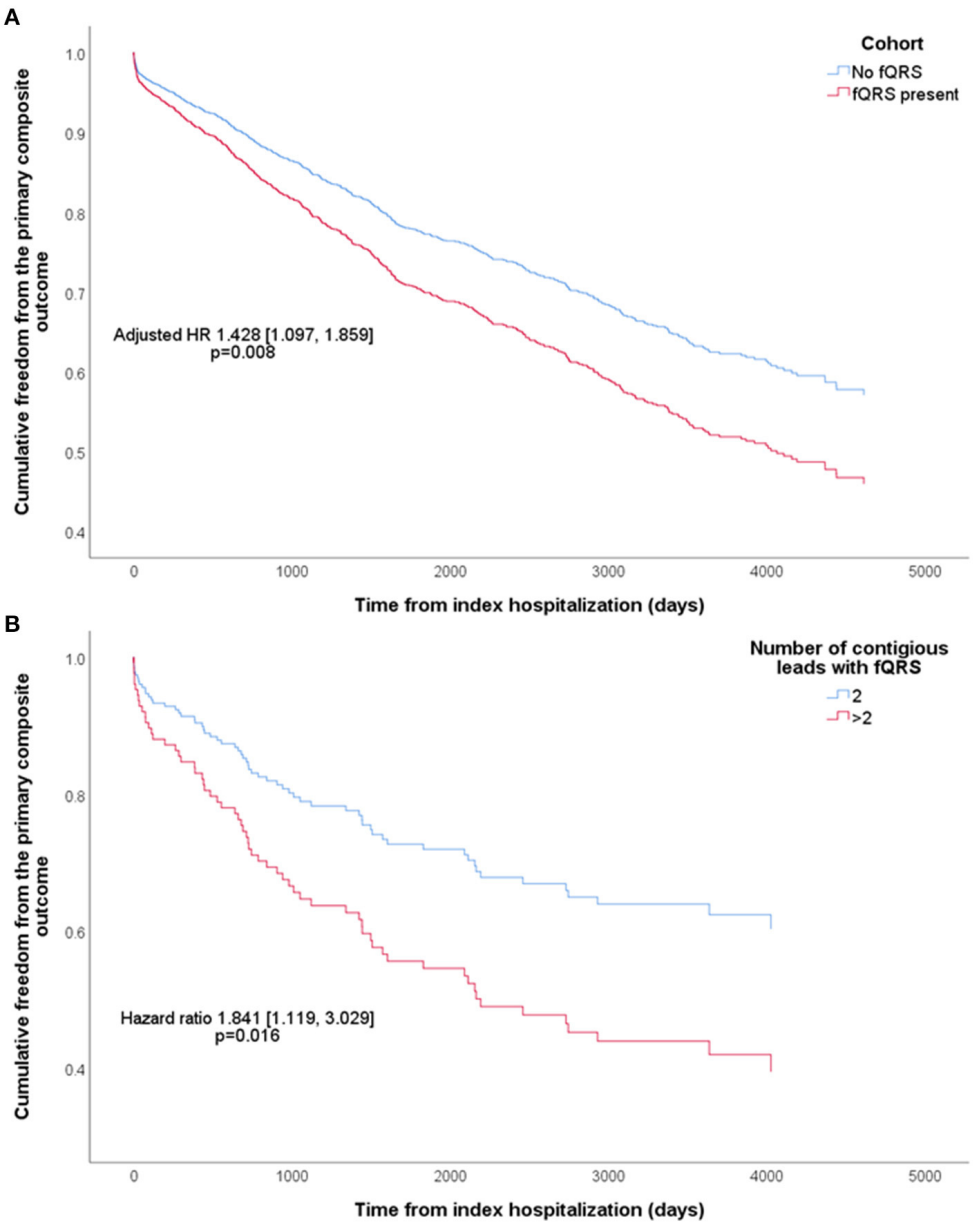
Kaplan-Meier curves of cumulative freedom from the primary composite outcome of **(A)** all patients, stratified by the presence of fragmented QRS (fQRS), and **(B)** patients with fQRS, stratified by fQRS burden. The hazard ratio (HR) shown was adjusted for baseline characteristics that predicted the primary composite outcome.

Cox regression (Table 2) showed that the presence of fQRS predicted the primary composite outcome in univariate analysis (HR 1.507 [1.160, 1.959], *p* = 0.002), which remained significant after multivariable adjustments (adjusted HR 1.428 [1.097, 1.859], *p* = 0.008; Figure 2A). Both univariate and multivariate analyses demonstrated that fQRS was strongly predictive of cardiovascular mortality, VA, and SCD. Importantly, the prognostic power of fQRS for the primary composite outcome, VA, and SCD were independent of prior history of VA and SCD.

**Table 2:** Cox regression analysis of all 2,182 patients.

**Outcome**		**Univariable**		**Multivariable model**	
		**HR [95% CI]**	***P*-value**	**HR [95% CI]**	***P*-value**
Composite primary outcome^a^		**1.507 [1.160, 1.959]**	**0.002**	**1.428 [1.097, 1.859]^b^**	**0.008**
Secondary outcomes	CV mortality	**1.373 [1.008, 1.871]**	**0.044**	**1.405 [1.030, 1.917]^c^**	**0.032**
	VA	**3.231 [1.707, 6.119]**	**0.0003**	**2.017 [1.039, 3.917]^d^**	**0.038**
	SCD	**1.832 [1.231, 2.727]**	**0.003**	**1.638 [1.097, 2.446]^e^**	**0.016**
	MI	1.023 [0.700, 1.496]	0.905	0.890 [0.607, 1.304]^f^	0.890
	New-onset AF	1.111 [0.874, 1.413]	0.391	1.123 [0.882, 1.431]^g^	0.347

*Hazard ratios were referenced against patients without fragmented QRS*.

a *A composite of cardiovascular mortality, ventricular arrhythmia, and sudden cardiac death*.

b *Adjusted for age, Charlson comorbidity index, anemia, chronic renal diseases, ischaemic heart disease, prior VA, prior SCD, prior MI, and diabetes mellitus*.

c *Adjusted for age, sex, anemia, Charlson comorbidity index, chronic renal disease, ischaemic heart disease, prior MI, and diabetes mellitus*.

d *Adjusted for age, sex, Charlson comorbidity index, anemia, ischaemic heart disease, prior MI, prior VA, and prior SCD*.

e *Adjusted for age, sex, Charlson comorbidity index, prior VA, prior SCD, and diabetes mellitus*.

f *Adjusted for age, sex, Charlson comorbidity index, known heart failure prior to index hospitalization, prior MI, ischaemic heart disease, diabetes mellitus, prior AF, and anemia*.

g *Adjusted for age, sex, Charlson comorbidity index, known heart failure prior to index hospitalization, prior MI, ischaemic heart disease, diabetes mellitus, and prior VA*.

*AF, atrial fibrillation; CI, confidence interval; CV, cardiovascular; HR, hazard ratio; MI, myocardial infarction; SCD, sudden cardiac death; VA, ventricular arrhythmia*.

*Statistically significant results were highlighted in bold*.

### Subgroup Analysis by IHD Status

Subgroup analysis was performed on patients with [786 (36.0%)] and without [1,396 (64.0%)] IHD (Supplementary Table 2). fQRS was present in 75 (9.5%) patients with IHD, and 104 (7.4%) patients without IHD. Patients with IHD were followed up for slightly longer periods (5.82 ± 4.13 vs. 5.29 ± 4.00 years, *p* = 0.003). Among patients with IHD, the primary composite outcome was met in 228 (29.0%) patients, while the outcome of cardiovascular mortality was met in 171 (21.8%) patients, VA in 28 (3.6%) patients, SCD in 83 (10.6%) patients, MI in 204 (26.0%) patients, and new-onset AF in 249 (31.7%) patients. Among patients without IHD, these were met in 338 (24.2%), 248 (17.8%), 28 (2.0%), 129 (9.2%), 149 (10.7%), and 580 (41.5%) patients, respectively. Cox regression showed that in patients with IHD, fQRS predicted the primary composite outcome (adjusted HR 1.511 [1.030, 2.215], *p* = 0.035), cardiovascular mortality (adjusted HR 1.615 [1.053, 2.479], *p* = 0.028), VA (adjusted HR 2.880 [1.160, 7.154], *p* = 0.023), and SCD (adjusted HR 1.881 [1.014, 3.481], *p* = 0.045). However, fQRS did not predict the primary composite outcome in patients without IHD (adjusted HR 1.342 [0.929, 1.938], *p* = 0.117), despite remaining strongly predictive of VA (adjusted HR 3.336 [1.343, 8.282], *p* = 0.009) and SCD (adjusted HR 1.928 [1.135, 3.278], *p* = 0.015).

### Prognostic Value of fQRS Burden

Patients with fQRS were stratified into those with two contiguous leads with fQRS (112 patients; 5.13% of all patients; 62.6% of patients with fQRS), and those with more than two such leads (67 patients; 3.07% of all patients; 37.4% of patients with fQRS), as summarized in Supplementary Table 3. The two subgroups were not significantly different in any baseline characteristics. Cox regression demonstrated significantly higher risk of the primary composite outcome in the latter (HR 1.841 [1.119, 3.029], *p* = 0.016; Figure 2B), driven by a significantly higher risk of SCD (HR 2.866 [1.350, 6.081], *p* = 0.006) which remained significant even after adjustment for clinical history of VA and SCD (HR 2.679 [1.252, 5.729], *p* = 0.011). All other outcomes were not significantly different in risk between the two subgroups.

## Discussion

In this retrospective cohort study of heart failure patients, fQRS is strongly and independently predictive of cardiovascular mortality, VA, and SCD, with the presence of fQRS in more than two contiguous leads being associated with significantly higher risk of SCD. Additionally, we showed that while fQRS was strongly predictive of VA and SCD regardless of IHD status, though fQRS was only predictive of cardiovascular mortality in patients with IHD.

Previous studies have shown that fQRS is predictive of mortality and arrhythmic events in patients with heart failure (4). To the authors' best knowledge, the present study is the first study focusing on Asian patients hospitalized with heart failure that reported long-term clinical outcomes, with a mean follow-up duration exceeding 5 years. We confirmed that the elevated risk of adverse outcomes in patients with fQRS persists in the long term, independent of prior history of VA. The magnitudes of elevated risk we observed were also comparable to previous studies (4). Previous studies on Asian patients with heart failure have found fQRS in 21.5–49% of patients (6, 10, 11). This wide range of prevalence was in spite of all these studies, including ours, using the same criteria set forth by Das et al. (5) It is thus likely that such variations were driven by the different inclusion criteria and, in turn, the severity and etiology of heart failure in included patients. For instance, Igarashi et al. reported the highest prevalence (49%) in a cohort of patients with ischaemic cardiomyopathy undergoing cardiac resynchronization therapy, contrasting the 21.5% reported by Pei et al. who analyzed patients with either dilated or ischaemic cardiomyopathy on optimal medical therapy (6, 10). In contrast, we identified fQRS in 8.20% of included patients. The lower rate was likely due to the less selective inclusion criteria of the current study, including patients regardless of previous heart failure diagnosis, admissions, and severity. Although this might introduce some heterogeneity, our study population closely reflect heart failure patients that clinicians see and manage on a daily basis. This strength was further reinforced by our use of a territory-wide database as data source. Overall, our results are useful for guiding clinicians in their care of patients with heart failure.

Additionally, we found that a higher burden of fQRS was associated with further increased risk of SCD. A similar concept has been shown by Debonnaire et al. in patients with hypertrophic cardiomyopathy (12). This finding may be explained mechanistically by higher fQRS burden reflecting more extensive myocardial scarring (13). By choosing a cutoff of two contiguous leads with fQRS, our results meant that the presence of additional leads with QRS complexes of fragmented morphology beyond the classification criteria would be additionally predictive of SCD. This allowed for a straightforward and intuitive interpretation, potentially facilitating clinical applications. The concept of fQRS burden was also echoed by Roudijk et al., who recently quantified fQRS burden by the total number of deflections in the QRS complex in all leads of a 12-lead ECG (14). Although the resultant index (quantitative fQRS, i.e., Q-fQRS) was not significantly prognostic in their studied population of arrhythmogenic cardiomyopathy, Q-fQRS may objectively provide more granularity in terms of fQRS burden than simpler, morphologically dichotomous approaches as used in the current study. The value of Q-fQRS deserves further studies and investigation in broader populations.

Importantly and interestingly, we showed that fQRS was predictive of SCD but not cardiovascular mortality in patients without IHD. It must be noted that non-ischaemic cardiomyopathies are a heterogeneous entity with various etiologies, and the pathophysiological changes leading to the presence of fQRS might vary accordingly. It is thus possible that fQRS has varying significance in these patients depending on the etiology of cardiomyopathy. Further studies are warranted in this area.

### Clinical Relevance

fQRS may serve as a simple yet powerful predictor of adverse outcomes in patients hospitalized for heart failure, facilitating risk stratification and management of such patients. For instance, the presence of fQRS may alert clinicians to arrange more intensive follow-ups and monitoring, especially for arrhythmia. A higher fQRS burden should also alert clinicians to a higher risk of VA and SCD. Further research of the applicability of fQRS in different patient population have the potential to influence decision pathways for primary and secondary prevention of arrhythmic events and SCD. Further investigation of the interactions between fQRS and other important echocardiographic and functional factors [e.g., frailty (15, 16)] may further impact the management of heart failure in general.

## Limitations

This study has several limitations. First, due to the nature of CDARS, we had no accompanying echocardiographic or functional data. We recognize that echocardiographic data, including left ventricular ejection fraction and other measures of systolic and diastolic function, is critical for prognosticating and classifying heart failure (17, 18). While the lack of these parameters limits interpretation, our findings remain clinically useful as ECG measurements are more readily available than echocardiographic measurements. We have also included the Charlson comorbidity index to better capture the patients' overall comorbid status. Second, the included patients with heart failure were heterogeneous in etiology and phenotype. As shown by the subgroup analysis on patients with or without IHD, such differences may affect the prognostic value of fQRS. The lack of information about non-ischaemic etiologies of heart failure in our cohort also limits the results' generalizability. Third, our analysis did not account for differences in the morphologies of fQRS, which may have prognostic implications (2). In addition to affecting the classification of fQRS, morphological considerations may also have implications in the quantification of fQRS burden, as discussed above. Although more detailed quantification of fQRS burden deserves further investigation (14), our results remain valid and clinically relevant given the strong prognostic values of fQRS burden as defined in this study. Fourth, data obtained from CDARS could not be adjudicated. However, all data entry were done by clinicians not involved in this study, and CDARS has been used extensively in other peer-reviewed publications (19, 20).

## Conclusion

The presence of fQRS was independently predictive of cardiovascular mortality, VA, and SCD in Asian patients hospitalized for heart failure. Having fQRS in more than two contiguous leads independently predicted further increased risk of SCD. However, fQRS did not predict cardiovascular mortality in patients without IHD, warranting further studies.

## Data Availability Statement

The raw data supporting the conclusions of this article will be made available by the authors, without undue reservation.

## Ethics Statement

The studies involving human participants were reviewed and approved by the Joint Chinese University of Hong Kong—New Territories East Cluster Clinical Research Ethics Committee. Written informed consent for participation was not required for this study in accordance with the national legislation and the institutional requirements.

## Author Contributions

JC and GT conceptualized this study. JZ, SL, AL, MT, and KL organized the database. JC performed the statistical analyses and wrote the first draft of the manuscript. GT wrote sections of the manuscript. KJ, TL, LR, YL, and QZ provided critical input. All authors contributed to manuscript revision, read, and approved the submitted version.

## Conflict of Interest

The authors declare that the research was conducted in the absence of any commercial or financial relationships that could be construed as a potential conflict of interest.

## Publisher's Note

All claims expressed in this article are solely those of the authors and do not necessarily represent those of their affiliated organizations, or those of the publisher, the editors and the reviewers. Any product that may be evaluated in this article, or claim that may be made by its manufacturer, is not guaranteed or endorsed by the publisher.

## References

[1] Boineau JP Cox JL. Slow ventricular activation in acute myocardial infarction. a source of re entrant premature ventricular contractions. Circulation. (1973) 48:702–13. 10.1161/01.CIR.48.4.702 4126756

[2] Haukilahti MAE Eranti A Kenttä T Huikuri H V. QRS fragmentation patterns representing myocardial scar need to be separated from benign normal variants: hypotheses and proposal for morphology based classification. Front Physiol. (2016) 7:653. 10.3389/fphys.2016.00653 28082919 PMC5183580

[3] Homsi M Alsayed L Safadi B Mahenthiran J Das MK. Fragmented QRS complexes on 12-lead ECG: a marker of cardiac sarcoidosis as detected by gadolinium cardiac magnetic resonance imaging. Ann Noninvasive Electrocardiol. (2009) 14:319–26. 10.1111/j.1542-474X.2009.00320.x 19804507 PMC6932232

[4] Kanitsoraphan C Rattanawong P Mekraksakit P Chongsathidkiet P Riangwiwat T Kanjanahattakij N. Baseline fragmented QRS is associated with increased all-cause mortality in heart failure with reduced ejection fraction: a systematic review and meta-analysis. Ann Noninvasive Electrocardiol. (2019) 24:e12597. 10.1111/anec.12597 30329201 PMC6931499

[5] Das MK Khan B Jacob S Kumar A Mahenthiran J. Significance of a fragmented QRS complex versus a Q wave in patients with coronary artery disease. Circulation. (2006) 113:2495–501. 10.1161/CIRCULATIONAHA.105.595892 16717150

[6] Pei J Li N Gao Y Wang Z Li X Zhang Y. The J wave and fragmented QRS complexes in inferior leads associated with sudden cardiac death in patients with chronic heart failure. Europace. (2012) 14:1180–7. 10.1093/europace/eur437 22308082

[7] Younis AS El-Halag MI ElBadry MA Abbas NIM. Fragmented QRS complex frequency and location as predictor of cardiogenic shock and mortality following acute coronary syndrome. Egypt Hear J. (2020) 72:43. 10.1186/s43044-020-00076-y 32705448 PMC7378133

[8] Rajadurai J Tse HF Wang CH Yang NI Zhou J Sim D. Understanding the epidemiology of heart failure to improve management practices: an asia-pacific perspective. J Card Fail. (2017) 23:327–39. 10.1016/j.cardfail.2017.01.004 28111226

[9] Martinez-Amezcua P Haque W Khera R Kanaya AM Sattar N Lam CSP. The upcoming epidemic of heart failure in South Asia. Circ Hear Fail. (2020) 13:e007218. 10.1161/CIRCHEARTFAILURE.120.007218 32962410

[10] Igarashi M Tada H Yamasaki H Kuroki K Ishizu T Seo Y. Fragmented QRS is a novel risk factor for ventricular arrhythmic events after receiving cardiac resynchronization therapy in nonischemic cardiomyopathy. J Cardiovasc Electrophysiol. (2017) 28:327–35. 10.1111/jce.13139 27925329

[11] Sha J Zhang S Tang M Chen K Zhao X Wang F. Fragmented QRS is associated with all-cause mortality and ventricular arrhythmias in patient with idiopathic dilated cardiomyopathy. Ann Noninvasive Electrocardiol. (2011) 16:270–5. 10.1111/j.1542-474X.2011.00442.x 21762255 PMC6932517

[12] Debonnaire P Katsanos S Joyce E Van Den Brink OVW Atsma DE Schalij MJ. QRS fragmentation and QTc duration relate to malignant ventricular tachyarrhythmias and sudden cardiac death in patients with hypertrophic cardiomyopathy. J Cardiovasc Electrophysiol. (2015) 26:547–55. 10.1111/jce.12629 25648421

[13] Konno T Hayashi K Fujino N Oka R Nomura A Nagata Y. Electrocardiographic QRS fragmentation as a marker for myocardial fibrosis in hypertrophic cardiomyopathy. J Cardiovasc Electrophysiol. (2015) 26:1081–7. 10.1111/jce.12742 26102305

[14] Roudijk RW Bosman LP Heijden JF van der Bakker JMT de Hauer RNW Tintelen JP van. Quantitative approach to fragmented QRS in arrhythmogenic cardiomyopathy: from disease towards asymptomatic carriers of pathogenic variants. J Clin Med. (2020) 9:545. 10.3390/jcm9020545 32079223 PMC7073517

[15] Zhang Y Yuan M Gong M Li G Liu T Tse G. Associations between prefrailty or frailty components and clinical outcomes in heart failure: a follow-up meta-analysis. J Am Med Dir Assoc. (2019) 20:509–10. 10.1016/j.jamda.2018.10.029 30541690

[16] Zhang Y Yuan M Gong M Tse G Li G Liu T. Frailty and clinical outcomes in heart failure: a systematic review and meta-analysis. J Am Med Dir Assoc. (2018) 19:1003–8.e1. 10.1016/j.jamda.2018.06.009 30076123

[17] Ponikowski P Voors AA Anker SD Bueno H Cleland JGF Coats AJS. 2016 ESC Guidelines for the diagnosis and treatment of acute and chronic heart failure. Eur Heart J. (2016) 37:2129–200. 10.1093/eurheartj/ehw128 27206819

[18] Chan JSK Tse G Zhao H Luo XX Jin CN Kam K. Echocardiography update for primary care physicians: a review. Hong Kong Med J. (2020) 26:44–55. 10.12809/hkmj198080 32051329

[19] Tse G Zhou J Lee S Wong W Li X Liu T. Relationship between angiotensin-converting enzyme inhibitors or angiotensin receptor blockers and COVID-19 incidence or severe disease. J Hypertens. (2021) 39:1717–24. 10.1097/HJH.0000000000002866 34188006

[20] Zhou J Tse G Lee S Liu T Cao Z Zeng D. Interaction effects between angiotensin-converting enzyme inhibitors or angiotensin receptor blockers and steroid or antiviral therapies in COVID-19: a population-based study. J Med Virol. (2021) 93:2635–41. 10.1002/jmv.26904 33638553 PMC8013691

