# Supplementary material for: Fragmented QRS Is Independently Predictive of Long-Term Adverse Clinical Outcomes in Asian Patients Hospitalized for Heart Failure: A Retrospective Cohort Study
Source: Front Cardiovasc Med. 2021 Nov 11;8:738417. doi: 10.3389/fcvm.2021.738417 (PMC8631899; doi:10.3389/fcvm.2021.738417)
Supplement: Supplementary file 1 [file Table_1.PDF]

Supplementary Table S1. ICD-9 codes for comorbidities.

Diabetes mellitus			250	250.01	250.02	250.03	250.1	250.11	250.12	250.13	250.2
	250.21	250.22	250.23	250.3	250.31	250.32	250.33	250.4	250.41	250.42	250.43
	250.5	250.51	250.52	250.53	250.6	250.61	250.62	250.63	250.7	250.71	250.72
	250.73										
Chronic renal diseases			582	582	582.1	582.2	582.4	582.8	582.81	582.89	582.9
	583	583	583.1	583.2	583.4	583.6	583.7	585	585.1	585.2	585.3
	585.4	585.5	585.6	585.9	586	588	588	588.1	588.8	588.81	588.89
	588.9										
Hypertension		401	401.1	401.9	402	402.01	402.1	402.11	402.9	402.91	403
	403.01	403.1	403.11	403.9	403.91	404	404.01	404.02	404.03	404.1	404.11
	404.12	404.13	404.9	404.91	404.92	404.93	405	405.01	405.09	405.1	405.11
	405.19	405.9	405.91	405.99	437.2						
Heart failure		428	428.1	428.2	428.2	428.21	428.22	428.23	428.3	428.3	428.31
	428.32	428.33	428.4	428.4	428.41	428.42	428.43	428.9	398.91	402.01	402.11
	402.91	404.01	404.03	404.11	404.13	404.91	404.93				
Atrial fibrillation			427.31	429.4							
Ventricular arrhythmias			427.1	427.4	427.41	427.42					
Sudden cardiac death			427.5	427.69	798	798.1	798.2				
Stroke/TIA		435	435.1	435.2	435.3	435.8	435.9	433.81	433.91	434	436
	437	437.1	433.31	433.01	434.01	434.1	434.11	434.9	434.91	437.2	437.3
	437.4	437.5	437.6	437.7	437.8	437.9	430	431	432	432.1	432.9
IHD	410	410.01	410.02	410.1	410.11	410.12	410.2	410.21	410.22	410.3	410.31
	410.32	410.4	410.41	410.42	410.5	410.51	410.52	410.6	410.61	410.62	410.7
	410.71	410.72	410.8	410.81	410.82	410.9	410.91	410.92	411	411.1	411.8
	411.81	411.89	412	413	413.1	413.9	414	414.01	414.02	414.03	414.04
	414.05	414.06	414.07	414.1	414.11	414.12	414.19	414.2	414.3	414.4	414.8
	414.9										
Myocardial infarction			410	410.0	410.1	410.2	410.3	410.4	410.5	410.6	410.7
	410.8	410.9	411	411.0	411.1	411.8	412				
Anaemia		280	280.0	280.1	280.8	280.9	281	281.0	281.1	281.2	281.3
	281.4	281.8	281.9	282.2	282.3	282.8	282.9	283.0	283.1	283.10	283.11
	283.19	283.2	283.9	284.0	284.01	284.09	284.1	284.11	284.12	284.19	284.81
	284.9	285	285.0	285.1	285.2	285.21	285.22	285.29	285.3	285.8	285.9

IHD, ischaemic heart disease. TIA, transient ischaemic attack.

Supplementary Table S2. Cox regression results, stratified by the presence of ischaemic heart disease. Hazard ratios (HR) were referenced against patients without fragmented QRS.

Subgroup	Outcome		Univariate		Multivariate	
			HR [95% CI]	p value	HR [95% CI]	p value
Ischaemic heart disease present (N=786)	Composite primary outcome [1]		**1.703 [1.166, 2.488]**	**0.006**	**1.511 [1.030, 2.215]** [2]	**0.035**
	Secondary outcomes	Cardiovascular mortality	**1.745 [1.142, 2.668]**	**0.010**	**1.615 [1.053, 2.479]** [3]	**0.028**
		Ventricular arrhythmia	**2.842 [1.152, 7.014]**	**0.023**	**2.880 [1.160, 7.154]** [4]	**0.023**
		Sudden cardiac death	1.831 [0.993, 3.376]	0.053	1.780 [1.051, 3.013] [5]	**0.032**
		Myocardial infarction	1.085 [0.684, 1.722]	0.728	1.013 [0.635, 1.615] [6]	0.958
		New-onset atrial fibrillation	1.072 [0.709, 1.620]	0.742	1.056 [0.696, 1.602] [7]	0.799
No ischaemic heart disease (N=1396)	Composite primary outcome [1]		1.334 [0.926, 1.920]	0.121	1.349 [0.934, 1.947] [2]	0.110
	Secondary outcomes	Cardiovascular mortality	1.062 [0.672, 1.678]	0.797	1.064 [0.671, 1.686] [3]	0.792
		Ventricular arrhythmia	**3.487 [1.414, 8.601]**	**0.007**	**3.526 [1.399, 8.887]** [4]	**0.008**
		Sudden cardiac death	**1.809 [1.071, 3.053]**	**0.027**	**1.873 [1.103, 3.181]** [5]	**0.020**
		Myocardial infarction	0.788 [0.402, 1.547]	0.489	0.690 [0.349, 1.362] [6]	0.285
		New-onset atrial fibrillation	1.168 [0.870, 1.570]	0.302	1.158 [0.860, 1.561] [7]	0.334

[1] A composite of cardiovascular mortality, ventricular arrhythmia, and sudden cardiac death

[2] Adjusted for age, Charlson comorbidity index, anaemia, chronic renal diseases, prior VA, prior SCD, prior MI, and diabetes mellitus

[3] Adjusted for age, sex, anaemia, Charlson comorbidity index, chronic renal disease, prior MI, and diabetes mellitus

[4] Adjusted for age, sex, Charlson comorbidity index, anaemia, prior MI, prior VA, and prior SCD

[5] Adjusted for age, sex, Charlson comorbidity index, prior VA, prior SCD, and diabetes mellitus

[6] Adjusted for age, sex, Charlson comorbidity index, known heart failure prior to index hospitalization, prior MI, diabetes mellitus, prior AF, and anaemia

[7] Adjusted for age, sex, Charlson comorbidity index, known heart failure prior to index hospitalization, prior MI, diabetes mellitus, and prior VA

CI, confidence interval.

Supplementary Table S3. Cox regression of the 179 patients with fragmented QRS. Hazard ratios were referenced against patients with fragmented QRS present in only two contiguous leads.

Outcome		Hazard ratio [95% confidence interval])	p value
Composite primary outcome [1]		**1.841 [1.119, 3.029]**	**0.016**
Secondary outcomes	Cardiovascular mortality	1.539 [0.850, 2.786]	0.155
	Ventricular arrhythmia	1.897 [0.611, 5.888]	0.268
	Sudden cardiac death	**2.866 [1.350, 6.081]**	**0.006**
	Myocardial infarction	1.746 [0.835, 3.649]	0.139
	New-onset atrial fibrillation	1.096 [0.676, 1.775]	0.710

[1] A composite of cardiovascular mortality, ventricular arrhythmia, and sudden cardiac death
